# A Prospective Comparative Non-randomized Study of Ketofol Versus Midazolam Sedation in Flexible Bronchoscopy

**DOI:** 10.7759/cureus.108690

**Published:** 2026-05-11

**Authors:** Soumyadeep Ghosh, Anurag Mondal, Arnab Bhattacharya, Namrata Chattopadhyay

**Affiliations:** 1 Respiratory Medicine and Critical Care, Santiniketan Medical College, Burdwan, IND; 2 Community and Family Medicine, All India Institute of Medical Sciences, Guwahati, Guwahati, IND; 3 Preventive and Social Medicine, All India Institute of Hygiene and Public Health, Kolkata, IND; 4 Anesthesiology, Sharanya Multispeciality Hospital, Burdwan, IND; 5 Pediatrics, Burdwan Medical College, Burdwan, IND

**Keywords:** desaturation, flexible bronchoscopy, ketofol, midazolam, procedural sedation, respiratory safety

## Abstract

Background

Flexible bronchoscopy requires reliable sedation that preserves respiratory drive while ensuring patient comfort and procedural efficiency. Midazolam, a benzodiazepine, is widely employed but is associated with respiratory depression, unpredictable recovery kinetics, and compromised oxygenation, particularly in patients with underlying pulmonary disease. Ketofol, a fixed-ratio admixture of ketamine and propofol, exploits the pharmacodynamic complementarity of its constituents: ketamine maintains airway reflexes and sympathetic tone, while propofol confers rapid-onset sedation and swift recovery. Head-to-head comparative evidence for ketofol versus midazolam in flexible bronchoscopy remains limited.

Methods

This prospective comparative non-randomized study enrolled 80 adult patients scheduled for flexible bronchoscopy at the Department of Respiratory Medicine, Santiniketan Medical College Hospital. Allocation to the ketofol group (n = 40) or the midazolam group (n = 40) was determined by the treating bronchoscopist in consultation with the attending anesthesiologist before the procedure, based on clinical judgment, anticipated airway tolerance, procedural requirements, comorbidity profile, and operator familiarity with the sedative regimen. A standardized pre-procedure assessment form was used, and the sedation choice was documented before bronchoscope insertion. Because of the observational nature of the study, no randomization, allocation concealment, or blinding was employed. Continuous monitoring included pulse oximetry, non-invasive blood pressure, and electrocardiography. Primary endpoints were the incidence of intraoperative cough events and desaturation episodes (SpO_2_ < 90%). Secondary endpoints encompassed sedation depth (Richmond Agitation-Sedation Scale (RASS)), physician and patient satisfaction (5-point Likert scale), procedure duration, recovery time, and supplemental dose requirements.

Results

Baseline demographic and clinical characteristics were comparable between groups. Ketofol was associated with fewer cough events (2.1 ± 1.0 vs. 5.2 ± 1.6; p < 0.001) and lower rates of oxygen desaturation (5% vs. 60%; p < 0.00001). Physician and patient satisfaction scores were higher with ketofol (4.6 and 4.5, respectively) compared with midazolam (3.0 and 3.1). Procedure duration (12 ± 3 vs. 18 ± 4 min) and recovery time (13 ± 3 vs. 33 ± 10 min) were shorter in the ketofol group. No serious adverse events were recorded in either arm.

Conclusion

In this prospective comparative non-randomized study, ketofol sedation was associated with lower rates of oxygen desaturation, reduced cough frequency, shorter recovery times, and higher physician and patient satisfaction compared with midazolam during flexible bronchoscopy. These findings suggest that ketofol may provide favorable procedural and respiratory outcomes in selected patients undergoing bronchoscopy. However, larger randomized controlled trials are required to further validate these observations and establish causality.

## Introduction

Flexible bronchoscopy has become one of the most frequently performed diagnostic and therapeutic pulmonary interventions in clinical practice, offering a minimally invasive means of visualizing the tracheobronchial tree, obtaining tissue samples, and managing endobronchial pathology [1]. The procedural success of flexible bronchoscopy is critically dependent on the adequacy of sedation: insufficient sedation precipitates patient distress, cough-related complications, and procedural discontinuation, whereas excessive sedation poses risks of respiratory depression, cardiovascular instability, and prolonged recovery [2].

Midazolam, a short-acting benzodiazepine, has historically constituted the cornerstone of sedation protocols for flexible bronchoscopy owing to its anxiolytic, amnestic, and muscle-relaxant properties [3]. However, its principal liability is dose-dependent respiratory depression, a concern of particular salience in the setting of flexible bronchoscopy, where the working channel of the bronchoscope occupies a significant fraction of the tracheal lumen and patients frequently present with compromised baseline pulmonary reserve [4]. Furthermore, midazolam exhibits considerable inter-individual pharmacokinetic variability, rendering precise titration challenging and recovery times difficult to predict [5].

Ketofol is a binary admixture of ketamine and propofol, typically formulated in a 1:3 or 1:4 ketamine-to-propofol volumetric ratio. The rationale underlying this combination resides in the pharmacodynamic synergy of its components: ketamine, an N-methyl-D-aspartate (NMDA) receptor antagonist, provides dissociative analgesia, preserves upper airway reflexes, and maintains bronchomotor tone, while simultaneously attenuating the cardiovascular depression and emergence phenomena that can complicate propofol administration [6]. Propofol, in turn, tempers the sympathomimetic and sialolytic properties of ketamine, contributing rapid and titratable sedation with swift recovery [7]. This complementarity has positioned ketofol as an attractive candidate sedative for procedures demanding maintenance of spontaneous ventilation.

Despite a growing body of evidence supporting ketofol for emergency procedural sedation and gastrointestinal endoscopy, comparative data specifically evaluating ketofol against midazolam in the context of flexible bronchoscopy remain sparse [8]. Most published comparisons have contrasted ketofol with propofol alone, leaving the question of its relative merits over the traditionally employed midazolam-based approach unresolved. The primary null hypothesis of the study was that there would be no significant difference between ketofol and midazolam sedation with respect to oxygen desaturation and intra-procedural cough events during flexible bronchoscopy. The alternative hypothesis was that ketofol sedation would be associated with improved respiratory and procedural outcomes compared with midazolam. The present study was therefore undertaken to compare the safety and efficacy of ketofol with midazolam sedation in adult patients undergoing flexible bronchoscopy, with particular reference to respiratory outcomes, procedural efficiency, and clinician and patient satisfaction.

## Materials and methods

Study design and setting

This prospective comparative non-randomized study was conducted in the Department of Respiratory Medicine at Santiniketan Medical College Hospital over a period of nine months, including six months of patient recruitment followed by three months of data analysis. The study protocol was reviewed and approved by the Institutional Ethics Committee, and the study was conducted in accordance with the principles of the Declaration of Helsinki and International Council for Harmonisation Good Clinical Practice guidelines. Written informed consent was obtained from all participants prior to enrollment. The study was registered with the Clinical Trials Registry of India (CTRI/2025/06/088799).

Study participants

Sample size estimation was based on the primary outcome of oxygen desaturation (SpO_2_ <90%) using a comparison of two proportions. Assuming a desaturation incidence of 40% in the midazolam group and 10% in the ketofol group, with a two-sided α error of 0.05 and power of 80%, the minimum required sample size was calculated to be 32 patients per group. To compensate for potential exclusions and incomplete data, 40 patients were enrolled in each study arm.

Consecutive adult patients aged ≥18 years undergoing elective flexible bronchoscopy and classified as American Society of Anesthesiologists (ASA) physical status I-III were included in the study.

Patients were excluded if they had known hypersensitivity to ketamine, propofol, or midazolam; significant renal impairment (serum creatinine >2 mg/dL); hepatic dysfunction (transaminases >2 times the upper limit of normal); severe respiratory failure (PaO_2_ <60 mmHg on room air, requirement of non-invasive ventilation, or invasive mechanical ventilation); hemodynamic instability (e.g., hypotension or arrhythmias); raised intracranial pressure or seizure disorder; chronic use of sedatives, opioids, or alcohol dependence; pregnancy or lactation; psychiatric illness impairing consent or cooperation; or ASA physical status ≥IV.

We used serum creatinine >2 mg/dL as a practical exclusion threshold for clinically significant renal impairment, because elevated creatinine reflects reduced renal function and renal dysfunction can prolong sedative effects, particularly with benzodiazepines.

Sedation protocol and group allocation

Allocation to the ketofol group (n = 40) or the midazolam group (n = 40) was determined by the treating bronchoscopist in consultation with the attending anesthesiologist before the procedure, based on clinical judgment, anticipated airway tolerance, procedural requirements, comorbidity profile, and operator familiarity with the sedative regimen. A standardized pre-procedure assessment form was used, and the sedation choice was documented before bronchoscope insertion. Because of the observational nature of the study, no randomization, allocation concealment, or blinding was employed.

The target sedation depth was a Richmond Agitation-Sedation Scale (RASS) score of -2 to -3, corresponding to light-to-moderate sedation while preserving spontaneous ventilation and airway reflexes.

In the ketofol group, patients received a ketamine-propofol admixture, which was prepared immediately prior to administration under aseptic conditions as a ketamine-propofol admixture in a 1:3 volumetric ratio. The preparation consisted of ketamine hydrochloride (50 mg/mL) and propofol (10 mg/mL) mixed in the same syringe. Specifically, 2 mL of ketamine (100 mg) was combined with 30 mL of propofol (300 mg), resulting in a final admixture volume of 32 mL. The solution was gently mixed to ensure homogeneity before intravenous administration. An initial dose of 0.05 mL/kg was administered intravenously, followed by supplemental doses of 0.025 mL/kg every two minutes as required to achieve adequate sedation. In the midazolam group, patients received an initial intravenous dose of 2.5 mg, followed by incremental doses of 1 mg every two minutes as clinically indicated.

All patients received supplemental oxygen via nasal cannula at 2-5 L/min. Topical anesthesia was achieved using 1% lidocaine applied to the vocal cords and tracheobronchial tree. An anesthesiologist was present throughout the procedure to monitor sedation and manage any adverse events.

Monitoring and data collection

Continuous monitoring included pulse oximetry (SpO_2_), non-invasive blood pressure, heart rate, and electrocardiography. Baseline parameters were recorded prior to sedation, followed by recordings at five-minute intervals during the procedure and recovery period.

Sedation depth was assessed using the RASS. Data were recorded on standardized case record forms and subsequently entered into a secure electronic database.

Outcome measures

The primary outcomes were the number of intraoperative cough episodes per patient, which was defined as any distinct cough or coughing burst occurring during bronchoscope insertion or manipulation requiring interruption, suctioning, or additional sedation/topical anesthesia, and the incidence of oxygen desaturation, defined as SpO₂ <90% for at least 10 seconds.

Secondary outcomes included sedation depth (RASS score), physician satisfaction, and patient comfort assessed using a 5-point Likert scale, total procedure duration, recovery time (defined as time to achieve an Aldrete score ≥9), number of supplemental sedative doses, and incidence of adverse events, including hypotension, bradycardia, and hypoxia.

Statistical analysis

Continuous variables are presented as mean ± standard deviation (SD), while categorical variables are expressed as frequencies and percentages. Intergroup comparisons for continuous variables were performed using the independent samples t-test. Categorical variables were analyzed using the chi-square test or Fisher’s exact test, as appropriate. A p-value <0.05 was considered statistically significant. Statistical analysis was performed using IBM SPSS Statistics for Windows, Version 26 (Released 2018; IBM Corp., Armonk, New York, United States).

Ethical considerations

The study was conducted in accordance with institutional ethical standards and the Declaration of Helsinki (2013 revision). Participant confidentiality was maintained by anonymizing all data. Participation was voluntary, and patients retained the right to withdraw at any point without affecting their clinical care.

## Results

Participant flow

**Figure 1:**
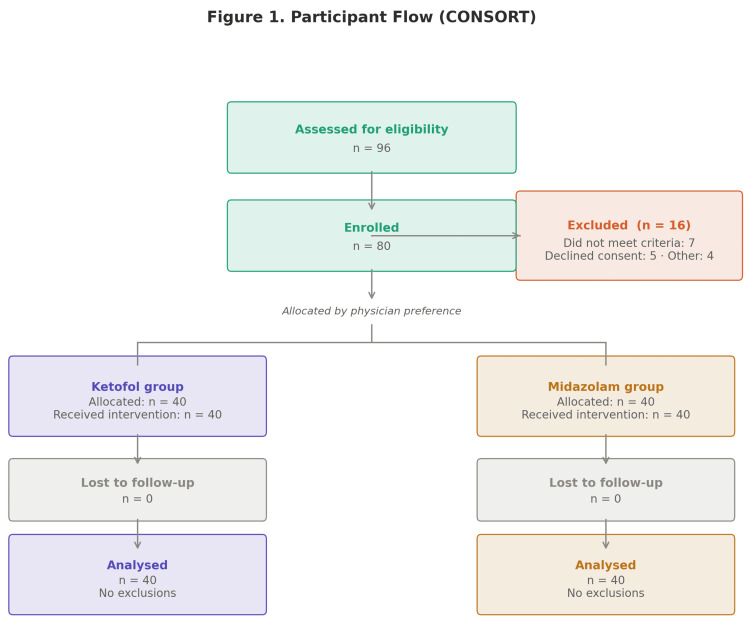
CONSORT flow diagram of participant selection and allocation CONSORT-style flow diagram depicting participant progression through the study phases. Of 96 patients screened, 16 were excluded (seven did not meet eligibility criteria, five declined participation, and four received alternative sedation). The remaining 80 patients were enrolled and allocated by physician preference to ketofol (n = 40) or midazolam (n = 40). All participants completed the study with no loss to follow-up and were included in the final analysis. CONSORT: Consolidated Standards of Reporting Trials

A total of 96 patients were assessed for eligibility. Of these, 16 (16.7%) were excluded: seven (7.3%) did not meet the inclusion criteria, five (5.2%) declined consent, and four (4.2%) received an alternative sedation regimen at the treating physician's discretion. The remaining 80 patients (83.3%) were enrolled and allocated to the ketofol group (n = 40 (50%)) or the midazolam group (n = 40 (50%)). No patients were lost to follow-up, and all 80 (100%) were included in the final analysis (Figure 1).

Baseline characteristics

Baseline demographic and clinical characteristics were comparable between groups (Table 1). In the ketofol group, the mean age was 52 ± 10 years, 24 patients (60%) were male, and seven (17.5%) had chronic obstructive pulmonary disease (COPD) or asthma. In the midazolam group, mean age was 53 ± 11 years, 23 patients (57.5%) were male, and 12 (30%) had COPD or asthma. Hypertension was present in 10 (25%) ketofol and 11 (27.5%) midazolam patients. Diabetes mellitus was identified in five (12.5%) and seven (17.5%) patients, respectively. Mean baseline SpO_2_ was 97 ± 1.2% in the ketofol group and 96 ± 1.4% in the midazolam group. None of these differences was statistically significant.

**Table 1:** Baseline demographic and clinical characteristics of the study population COPD: chronic obstructive pulmonary disease

Variable	Ketofol (n = 40)	Midazolam (n = 40)
Age (years, mean ± SD)	52 ± 10	53 ± 11
Male sex, n (%)	24 (60)	23 (57.5)
Hypertension, n (%)	10 (25)	11 (27.5)
Diabetes mellitus, n (%)	5 (12.5)	7 (17.5)
COPD/asthma, n (%)	7 (17.5)	12 (30)
Baseline SpO_2_ (%, mean ± SD)	97 ± 1.2	96 ± 1.4

Primary outcomes

Cough Episodes

**Figure 2:**
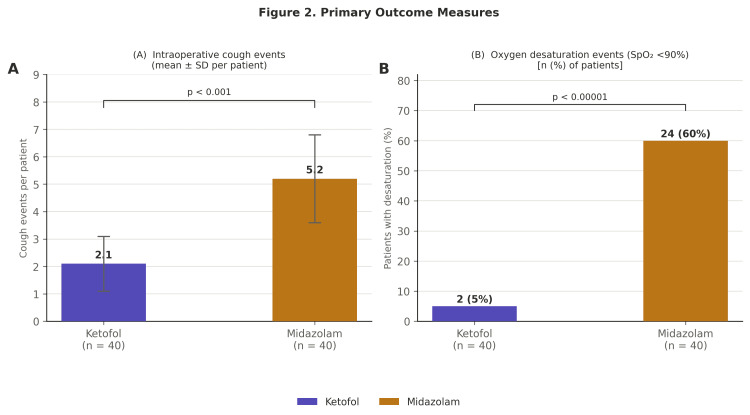
Primary outcome measures comparing ketofol and midazolam sedation (A) Mean number of intraoperative cough events per patient (mean ± SD). (B) Incidence of oxygen desaturation (SpO_2_ <90%). Ketofol demonstrated significantly fewer cough events and lower desaturation rates.

Patients in the ketofol group experienced fewer intraoperative cough events per patient (mean ± SD: 2.1 ± 1.0) compared with those receiving midazolam (5.2 ± 1.6; p < 0.001) (Figure 2A and Table 2). Oxygen desaturation (SpO_2_ <90%) occurred in two of 40 patients (2, 5%) in the ketofol group compared with 24 of 40 patients (24, 60%) in the midazolam group (p < 0.00001) (Figure 2B). These findings suggest an association between ketofol sedation and lower rates of cough and oxygen desaturation during flexible bronchoscopy.

**Table 2:** Primary outcome measures

Outcome	Ketofol (n = 40)	Midazolam (n = 40)	p-value
Cough events (mean ± SD)	2.1 ± 1.0	5.2 ± 1.6	<0.001
Desaturation events, SpO_2_ < 90%, n (%)	2 (5)	24 (60)	<0.00001

Secondary outcomes

Sedation depth, as measured by mean RASS score, was comparable between groups (-2.1 in the ketofol arm versus -2.3 in the midazolam arm), confirming that equivalent sedation depth was achieved with both regimens (Table 3).

**Table 3:** Secondary outcome measures RASS: Richmond Agitation-Sedation Scale

Outcome	Ketofol (n = 40)	Midazolam (n = 40)
RASS score (mean)	-2.1	-2.3
Physician satisfaction score (1-5)	4.6	3.0
Patient comfort score (1-5)	4.5	3.1
Procedure duration (min, mean ± SD)	12 ± 3	18 ± 4
Recovery time (min, mean ± SD)	13 ± 3	33 ± 10
Supplemental doses required (mean)	1.1	2.4

**Figure 3:**
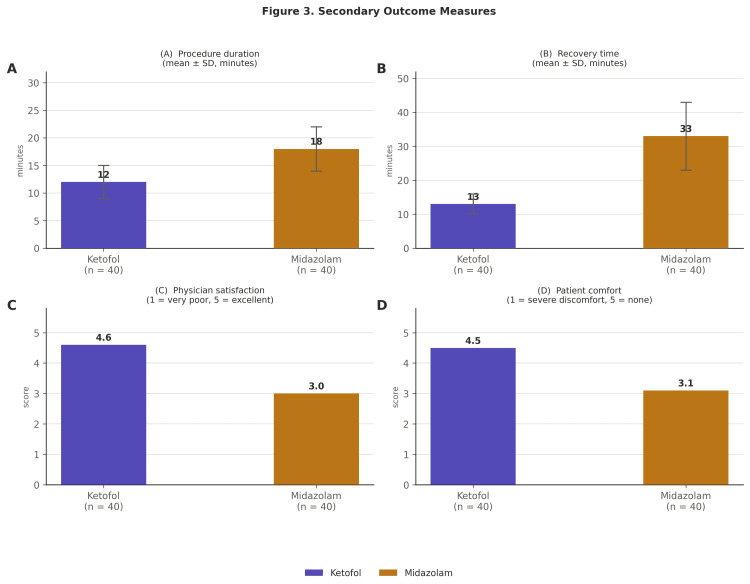
Secondary outcome measures comparing ketofol and midazolam sedation (A) Procedure duration (minutes, mean ± SD): ketofol 12 ± 3 vs. midazolam 18 ± 4. (B) Recovery time (minutes, mean ± SD): ketofol 13 ± 3 vs. midazolam 33 ± 10. (C) Physician satisfaction score (5-point Likert scale; 1 = very poor, 5 = excellent): ketofol 4.6 vs. midazolam 3.0. (D) Patient comfort score (5-point Likert scale; 1 = severe discomfort, 5 = none): ketofol 4.5 vs. midazolam 3.1.

Procedure duration was shorter in the ketofol group (12 ± 3 min) than in the midazolam group (18 ± 4 min) (Figure 3A). Recovery time was reduced with ketofol, with patients achieving an Aldrete Score ≥9 in a mean of 13 ± 3 min compared with 33 ± 10 min in the midazolam group (Figure 3B). The mean number of supplemental sedative doses required was lower with ketofol (1.1 doses vs. 2.4 doses).

Physician satisfaction was higher with ketofol, with a mean score of 4.6 on the 5-point scale versus 3.0 for midazolam (Figure 3C). Similarly, patient comfort was rated higher in the ketofol group (mean score 4.5 vs. 3.1) (Figure 3D), reflecting better tolerance of the procedure.

Adverse events

**Figure 4:**
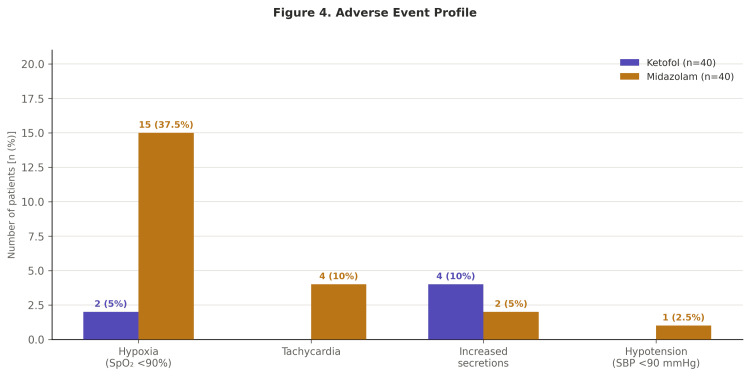
Adverse event profile in ketofol and midazolam groups Comparison of hypoxia, tachycardia, increased secretions, and hypotension between groups. No serious adverse events occurred in either group. SBP: systolic blood pressure

Post-procedural hypoxia (SpO_2_ < 90%) requiring oxygen supplementation was the most frequently recorded adverse event, occurring in two of 40 ketofol patients (2, 5%) compared with 15 of 40 midazolam patients (15, 37.5%). Tachycardia was recorded in four midazolam patients (4, 10%) and in none of the ketofol patients (0, 0%). Increased secretions attributable to ketamine’s sialogogue effect were noted in four ketofol patients (4, 10%) compared with two midazolam patients (2, 5%); all were managed with simple suctioning without procedural interruption. One midazolam patient (1, 2.5%) experienced transient hypotension (systolic blood pressure (SBP) < 90 mmHg) requiring brief observation; no ketofol patient developed hypotension (0, 0%). No serious adverse events - defined as death, life-threatening complications, or unplanned hospitalization - were recorded in either group (Figure 4).

## Discussion

The present prospective comparative non-randomized study demonstrates that ketofol sedation in flexible bronchoscopy may be associated with improved respiratory safety, procedural efficiency, and physician and patient satisfaction compared with midazolam. The incidence of oxygen desaturation was lower in the ketofol group compared with midazolam (5% vs. 60%), a difference that is clinically relevant, although it should be interpreted cautiously in light of the study design. The relatively high intra-procedural desaturation rate observed in the midazolam group warrants cautious interpretation. Several factors may have contributed to this finding, including the underlying pulmonary disease burden of the study population, bronchoscope-related airway narrowing during the procedure, and the respiratory depressant effects of benzodiazepine-based sedation. Notably, the midazolam group included a higher proportion of patients with COPD/asthma than the ketofol group, which may have increased susceptibility to oxygen desaturation during bronchoscopy. In addition, pulse oximetry rather than routine end-tidal CO_2_ monitoring was used during the study period, which may have limited early detection of hypoventilation. Although all patients received standardized supplemental oxygen and continuous monitoring, the observational single-center design and modest sample size necessitate cautious interpretation of these findings.

The evolution of sedation practices in flexible bronchoscopy reflects a broader shift toward optimizing the balance between adequate sedation and preservation of physiological function. Early practice predominantly relied on midazolam monotherapy due to its anxiolytic and amnestic properties and reversibility with flumazenil [9]. However, its dose-dependent respiratory depression and variable pharmacokinetics remain important limitations, particularly in patients with compromised pulmonary reserve.

Comparative evidence has progressively challenged the primacy of midazolam. Wang et al. demonstrated that propofol is associated with faster recovery compared to midazolam, with comparable safety profiles [10]. Similarly, Clark et al., in a randomized trial, reported improved patient cooperation and shorter recovery times with propofol compared to midazolam for flexible bronchoscopy sedation [11]. However, propofol requires careful titration due to its dose-dependent hemodynamic and respiratory depression risks [12]. These findings underscore the need for sedation strategies that maintain respiratory stability while ensuring procedural efficacy.

In this context, ketofol has emerged as a rational pharmacological combination. The observed reduction in desaturation events aligns with previous literature. Ebru and Resul demonstrated fewer desaturation episodes and reduced analgesic requirements with ketofol compared to midazolam in endoscopic retrograde cholangiopancreatography (ERCP) procedures [13], while Dal et al. concluded that ketofol was effective and safe for sedation in the endobronchial ultrasound-guided needle aspiration procedure [14]. Parallel investigations in emergency procedural sedation have also suggested that ketofol reduces clinically significant respiratory adverse events compared with propofol alone, while maintaining comparable procedural success and patient satisfaction [7,15].

Findings from bronchoscopy-specific literature further support these observations. Pertzov et al. demonstrated comparable respiratory outcomes between dexmedetomidine and propofol but noted a higher rate of adverse events with dexmedetomidine [16], highlighting the need for sedation strategies that balance both respiratory and cardiovascular safety.

The observed findings are consistent with the complementary pharmacodynamics of ketofol. Ketamine preserves airway reflexes, maintains respiratory drive, and provides bronchodilation via catecholamine-mediated mechanisms, thereby supporting oxygenation during the procedure. Propofol contributes rapid, titratable sedation, while its respiratory depressant effects are mitigated within the combination [17]. In contrast, midazolam causes dose-dependent respiratory depression through gamma-aminobutyric acid (GABA)-mediated central suppression, reducing hypoxic and hypercapnic ventilatory responses, particularly in patients with underlying pulmonary disease [18].

The lower cough frequency in the ketofol group represents another important clinical advantage. Excessive coughing can compromise visualization, prolong procedures, and increase complication risk. This reduction may be attributed to ketamine’s modulation of airway reflexes and bronchodilatory effects [19], which likely contributed to shorter procedure duration.

Procedure duration and recovery time were both significantly reduced with ketofol. These findings are consistent with prior reports highlighting the rapid pharmacokinetics of propofol compared with midazolam, which undergoes hepatic metabolism with active metabolites that may prolong sedation [20]. Faster recovery improves patient safety and enhances procedural outcomes. Higher physician and patient satisfaction scores further suggest that ketofol may offer practical advantages in procedural bronchoscopy, likely reflecting fewer hypoxic interruptions and smoother recovery.

Adverse event profiles were broadly comparable, although increased secretions were more frequently observed in the ketofol group, consistent with ketamine’s known sialagogue effect. This was not clinically significant and was easily managed with suctioning [21].

Several limitations of this study should be acknowledged. First, the observational design with physician-preference-based allocation introduces the possibility of selection bias and confounding by indication. Although baseline characteristics were broadly comparable, we cannot exclude the possibility that ketofol was preferentially selected for patients perceived to be more stable or cooperative. Second, the lack of blinding may have influenced cough counting, satisfaction scores, and the management of desaturation events. Third, sedation depth was assessed using the RASS, which is a clinically useful but subjective measure; an objective monitor such as bispectral index was not used. In addition, the absence of routine capnography during the study period is an important limitation, as reliance on pulse oximetry alone may delay recognition of early hypoventilation and underestimate subtle respiratory compromise. Finally, the single-center setting, modest sample size, and lack of pharmacoeconomic analysis may limit the generalizability and broader applicability of the findings.

Future research should focus on randomized controlled trials with larger sample sizes and stratification by baseline pulmonary function. Further studies exploring optimal ketamine-propofol ratios, the role of anticholinergic premedication, and cost-effectiveness analyses would provide valuable insights for clinical practice.

## Conclusions

This prospective comparative non-randomized study found that ketofol sedation was associated with lower rates of oxygen desaturation, reduced intraoperative cough frequency, shorter recovery times, and higher clinician and patient satisfaction compared with midazolam during flexible bronchoscopy. The pharmacodynamic complementarity of ketamine and propofol, combining preservation of respiratory drive with rapid-onset and titratable sedation, may make ketofol a useful option in bronchoscopy sedation practice. However, given the single-center non-randomized design and potential for allocation bias, these findings should be interpreted cautiously. Larger randomized controlled trials are warranted to further evaluate the safety and efficacy of ketofol across diverse patient populations and procedural settings.
